# FAIM-L regulation of XIAP degradation modulates Synaptic Long-Term Depression and Axon Degeneration

**DOI:** 10.1038/srep35775

**Published:** 2016-10-21

**Authors:** Ramón Martínez-Mármol, Bruna Barneda-Zahonero, David Soto, Rosa Maria Andrés, Elena Coccia, Xavier Gasull, Laura Planells-Ferrer, Rana S. Moubarak, Eduardo Soriano, Joan X. Comella

**Affiliations:** 1 Department of Cell Biology, University of Barcelona, 08028 Barcelona, Spain; 2 Centro de Investigación Biomédica en Red sobre Enfermedades Neurodegenerativas, Spain; 3 Institut de Neurociències, Departament de Bioquímica i Biologia Molecular, Facultat de Medicina, Universitat Autònoma de Barcelona, 08193 Bellaterra, Spain; 4 Institut de Recerca de l’Hospital Universitari de la Vall d’Hebron (VHIR), 08035 Barcelona, Spain; 5 Neurophysiology Laboratory, Physiology Unit, Department of Biomedicine, Medical School, Universitat de Barcelona, Barcelona, Spain; 6 Institute of Neurosciences, University of Barcelona, Barcelona, Spain.; 7 Institut d’Investigacions Biomèdiques August Pi i Sunyer (IDIBAPS), Barcelona, Spain; 8 ICREA Academia, 08010 Barcelona, Spain

## Abstract

Caspases have recently emerged as key regulators of axonal pruning and degeneration and of long-term depression (LTD), a long-lasting form of synaptic plasticity. However, the mechanism underlying these functions remains unclear. In this context, XIAP has been shown to modulate these processes. The neuron-specific form of FAIM protein (FAIM-L) is a death receptor antagonist that stabilizes XIAP protein levels, thus preventing death receptor-induced neuronal apoptosis. Here we show that FAIM-L modulates synaptic transmission, prevents chemical-LTD induction in hippocampal neurons, and thwarts axon degeneration after nerve growth factor (NGF) withdrawal. Additionally, we demonstrate that the participation of FAIM-L in these two processes is dependent on its capacity to stabilize XIAP protein levels. Our data reveal FAIM-L as a regulator of axonal degeneration and synaptic plasticity.

The long form of Fas Apoptotic Inhibitory Molecule (FAIM) protein, FAIM-L, expressed solely in neurons, is a Death Receptor (DR) antagonist that protects neurons from DR-induced apoptosis^1^. We recently reported the mechanism through which FAIM-L safeguards neurons from Fas Ligand (FasL)-induced apoptosis. In this regard, FAIM-L interacts with XIAP through an IAP-Binding Motif (IBM) located at its N-terminus. This association impairs XIAP auto-ubiquitinylation and degradation by the proteasome, hence enabling XIAP to confer protection against FasL-induced apoptosis by inhibiting caspase-3 activation^2^. Accordingly, we addressed whether XIAP stabilization by FAIM-L, leading to precise regulation of caspase-3, is involved not only in neuronal apoptosis but also in non-apoptotic physiological functions of XIAP in neurons.

During nervous system development, once neurons are integrated into circuits, apoptotic signaling is restricted to specific cellular compartments in order to maintain neuronal viability. Neural connections form a dynamic system, and many neurological processes participate in its final configuration and remodeling, such as axon-selective pruning or long-term regulation of synaptic plasticity. In this context, the apoptotic machinery has emerged as a critical component of these processes. In recent years, caspases-3, -6, -7 and -9 have been implicated either in axon degeneration^3^,^4^,^5^ and/or the regulation of neuronal synaptic plasticity^6^,^7^,^8^.

X-linked inhibitor of apoptosis protein (XIAP) tightly regulates caspase activation. XIAP interacts with the effector caspases-3 and -7 through the baculovirus inhibitor repeat (BIR) 2 domain^9^,^10^,^11^ and with the activator caspase-9 through the BIR3 domain^12^. The XIAP-caspase interaction inhibits the catalytic activity of caspases, thus modulating their cellular functions. Indeed, XIAP is involved in the regulation of the non-apoptotic functions of caspases. It controls caspase activity in degenerating axons^13^ and once the axon is degenerating, XIAP is also responsible for restricting caspase activation to this specific subcellular compartment^4^. XIAP has also been linked to the control of synaptic plasticity, thus its regulation of caspase-3, -7 and -9 activation modulates long-term depression (LTD)-induced AMPA receptor (AMPAR) internalization^6^. However, the contribution of FAIM-L-mediated XIAP stabilization to these non-apoptotic functions of XIAP has not been explored.

Here we examined the relevance of FAIM-L-mediated XIAP stabilization in two critical non-apoptotic processes, namely axon degeneration and synaptic plasticity. We report that the precise regulation of XIAP protein levels by FAIM-L affects the synaptic plasticity of hippocampal neurons exposed to NMDA-induced LTD, as well as the axon degeneration of dorsal root ganglion neurons (DRGs) induced by NGF withdrawal. Our data suggest that FAIM-L plays a key role in the maintenance of life-long neuronal plasticity by regulating axon degeneration and synaptic plasticity.

## Results

### FAIM-L modulates XIAP protein levels in hippocampal neurons *in vitro*

**Figure 1:**
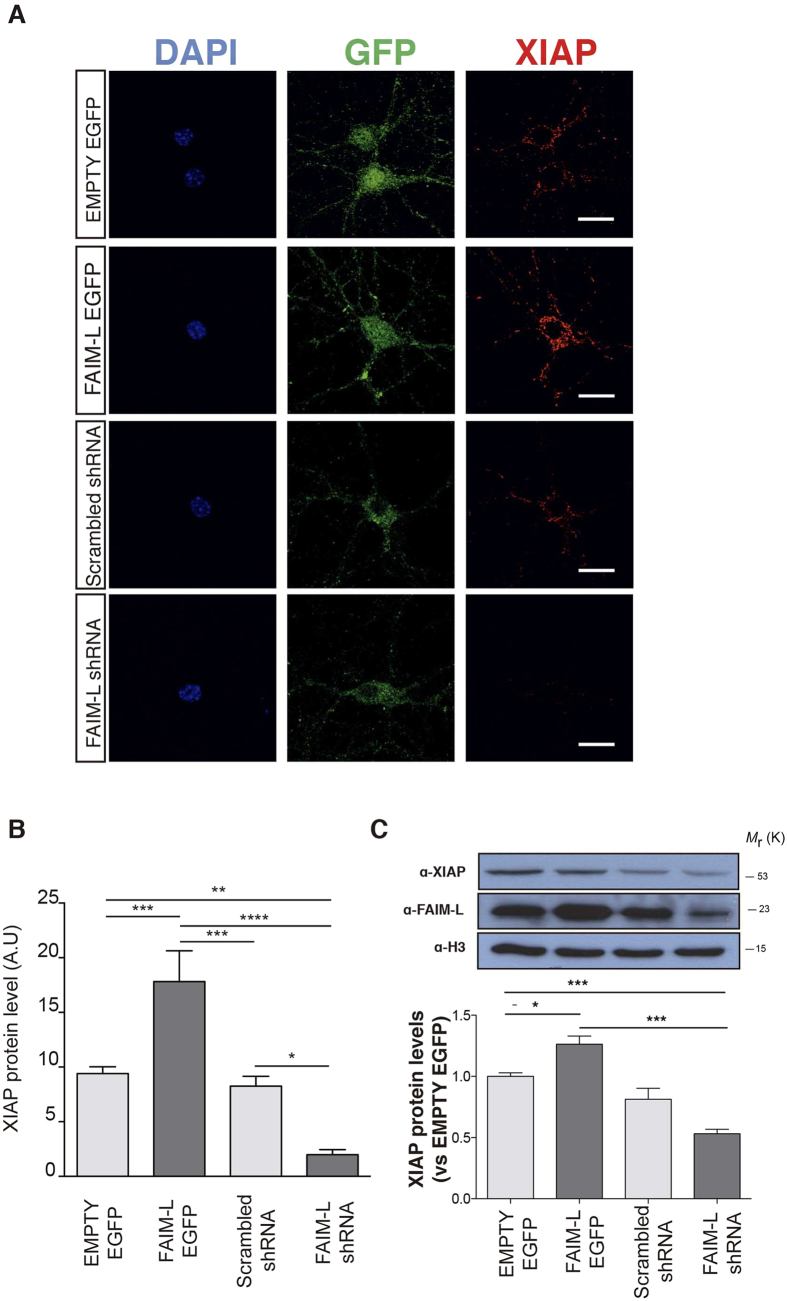
FAIM-L modulates the stability of XIAP protein levels in hippocampal neurons. (**A**) Confocal images of hippocampal neurons (15 DIV) infected with lentivirus containing EMPTY-EGFP, FAIM-L-EGFP, FAIM-L-shRNA or scrambled shRNA and immunolabeled against GFP (second column, green) and XIAP (third column, red). The scale bar represents 20 μm. (**B**) Quantification of XIAP staining relative to neuronal surface. A.U represent pixel intensities/μm^2^. N = 15 to 25 neurons from 3 independent experiments, for each group. Data represent mean ± SEM and One-way ANOVA followed by Tukey’s multiple comparison *post-hoc* test was used to calculate significant levels between the indicated groups. *p < 0.05; **p < 0.01; ***P < 0.001; ****p < 0.0001. (**C**) Hippocampal neurons were infected with lentiviral vectors as indicated. Protein levels of XIAP and FAIM-L were determined by Western blot 48 h after infection with overexpressing and silencing vectors. Data are represented as the mean ± standard error of the mean (SEM) of three independent experiments. One-way ANOVA followed by Tukey’s multiple comparison *post-hoc* test was used to calculate significant levels between the indicated groups. *p < 0.05; **p < 0.01.

Mitochondria engagement, and caspase-3 activation and its modulation by XIAP participate in LTD regulation, a crucial physiological process for CNS development and homeostasis^6^,^7^,^8^. Our previous data revealed the capacity of the DR antagonist, FAIM-L, to stabilize XIAP levels, and thus, to protect cortical neurons from FasL-induced cell death^2^. Therefore, we first assessed the capacity of FAIM-L to regulate XIAP levels in hippocampal neurons. Cultured neurons were transduced with FAIM-L or empty EGFP-tagged lentiviral vectors for overexpression and shRNA-FAIM-L or shRNA-scrambled for gene silencing. XIAP expression was assessed by immunocytochemistry and Western blot. The overexpression or silencing of FAIM-L in hippocampal neurons resulted in the up-regulation or the abolishment of XIAP expression, respectively (Fig. 1A–C).

### FAIM-L regulates AMPAR internalization after NMDA-induced LTD in hippocampal neurons

**Figure 2:**
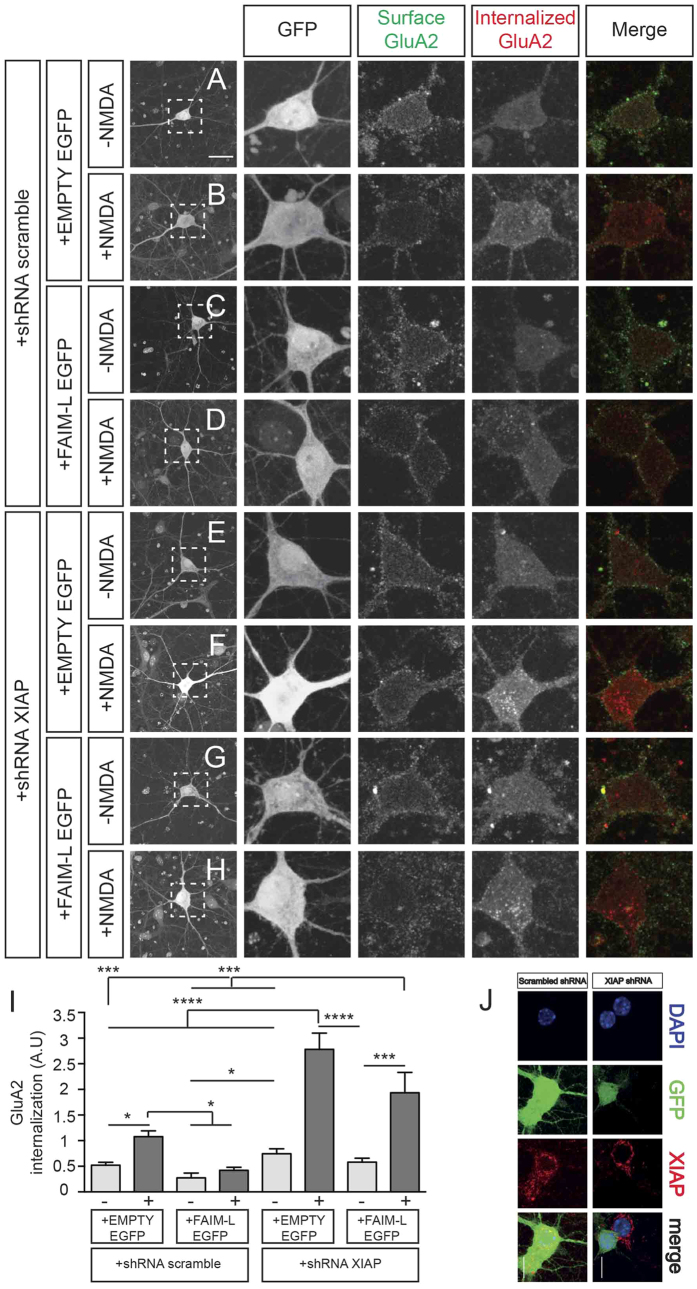
FAIM-L affects NMDA-induced AMPA receptor internalization in hippocampal neurons by inducing endogenous XIAP stabilization. Antibody-feeding internalization assay for endogenous GluA2 in hippocampal neurons stimulated with NMDA (50 μM for 15 min, (**B**,**D**,**F**,**H**). Neurons were previously infected with lentivirus containing with the shRNA vectors (scrambled –**A**,**B**,**C**,**D**– or XIAP –**E**,**F**,**G**,**H**–) and after 48 h were re-infected with either EMPTY-EGFP (**A**,**B**,**E**,**F**) or FAIM-L-EGFP (**C**,**D**,**G**,**H**) vectors, as indicated. The figure shows triple-label immunostaining for infected GFP-positive neurons (first and second column), surface-remaining GluA2 (third column, green in merge), internalized GluA2 (fourth column, red in merge), and merge (fifth column). Individual channels are shown in gray scale. Images in columns 2, 3, 4 and 5 represent magnifications from selected areas of the first columns. The scale bar represents 20 μm. (**I**) Shows quantitation of internalization index for the experiment represented in (**A–H**) integrated fluorescence intensity of internalized GluA2/integrated fluorescence intensity of surface-remaining GluA2. Results were not normalized to untreated cells (−NMDA)^6^. As a consequence, A.U have no dimensions. N = 55 to 67 neurons from 3 independent experiments, for each group. Data represent mean ± SEM and and One-way ANOVA test followed by Tukey’s multiple comparison *post-hoc* test was used to calculate significant levels between the indicated groups. *p < 0.05; ***P < 0.001; ****p < 0.0001. (**J**) control experiment to demonstrate that the used XIAP-shRNA efficiently down-regulates the expression of its target protein. Hippocampal neurons were infected with lentivirus containing the scramble-shRNA vectors (upper panels) or XIAP-shRNA vectors (lower panels) and 72 h later they were fixed and immune-stained against XIAP. In lower panels, it can be clearly appreciated that only neurons infected with XIAP-shRNA showed a clear reduction of XIAP protein levels. However, the XIAP protein levels are not affected in non-infected neurons or neurons infected with scramble-shRNA.

**Figure 3:**
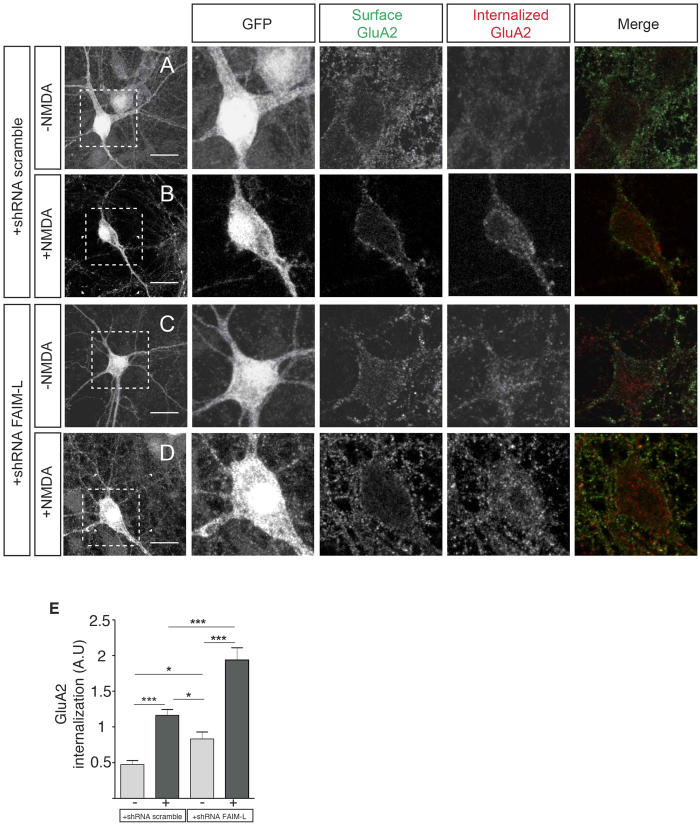
FAIM-L actively participates in GluA2 internalization induced by chemical LTD. (**A**–**D**) antibody-feeding internalization assay for endogenous GluA2 in hippocampal neurons stimulated with NMDA (50 μM for 15 min). Neurons were infected with lentiviruses containing the shRNA constructs (scrambled or FAIM-L). The figure shows triple-label immunostaining for infected GFP-positive neurons (first and second column), surface-remaining GluA2 (third column, green in merge), internalized GluA2 (fourth column, red in merge), and merge (fifth column). Individual channels are shown in grayscale. Images in columns 2, 3, 4 and 5 represent magnifications from selected areas of the first columns. The scale bar represents 20 μm. (**E**) quantification of GluA2 internalization in the indicated conditions calculated as in Fig. 2(I). Results were not normalized to untreated cells (−NMDA). As a consequence, A.U have no dimensions. N = 48 to 53 neurons from 3 independent experiments, for each group. Data represent means ± SEM and were analyzed by the One-way ANOVA test followed by Newman-Keuls multiple comparison *post-hoc* test, ***p < 0.001, *p < 0.05.

Li and collaborators reported that XIAP and other members of the intrinsic apoptotic pathway regulate the internalization of the AMPAR subunit GluA2 after chemical-LTD induction. They proved that XIAP overexpression blocks NMDA-induced GluA2 internalization not only in hippocampal cultures, but also in CA1 organotypic slice cultures, where XIAP overexpression abrogated the LTD induced by low frequency stimulation^6^. Since we observed that FAIM-L regulates XIAP protein stability in hippocampal neurons, we next assessed its relevance in LTD. To address this issue, we used an *in vitro* model of LTD (chemical-LTD) that consists of NMDA treatment of hippocampal neurons for 15 min. The induction of LTD is monitored by the internalization of AMPAR subunits, such as GluA1 and GluA2. AMPAR endocytosis was measured in dissociated hippocampal neurons by means of an “antibody feeding” internalization assay^14^. Hippocampal neurons were infected with FAIM-L or empty EGFP-Tagged lentiviruses for overexpression, together with shRNA-XIAP, shRNA-FAIM-L or shRNA-scrambled for gene silencing (Figs 2 and 3). At 15–18 DIV, cultures were treated with 50 μM NMDA for 15 min at 37 °C to stimulate chemical-LTD-induced GluA2 internalization. In control conditions (see +shRNA-scrambled, +EMPTY-EGFP; Fig. 2A,B,I), the internalization was reduced when FAIM-L was overexpressed, as no significant differences were observed when comparing GluA2 internalized levels after treatment with control medium or NMDA in this condition (see +shRNA-scrambled, +FAIML-EGFP; Fig. 2C,D,I). In agreement with the observations of Li *et al.*^6^, we found that NMDA-induced GluA2 internalization was greatly increased when XIAP was knocked down using specific shRNA (see +shRNA-XIAP, +EMPTY-EGFP; Fig. 2E,F,I). Moreover, the reduction of GluA2 internalization caused by FAIM-L overexpression was not detected after XIAP silencing, thus further strengthening the notion that FAIM-L exerts its effect through the stabilization of XIAP protein (see +shRNA-XIAP, +FAIM-L-EGFP; Fig. 2G,H,I). The efficacy of shRNA-XIAP on XIAP expression was tested in Fig. 2J.

Finally, we tested the participation of endogenous FAIM-L in regulating the internalization of AMPAR subunit GluA2. With this aim, we examined the levels of GluA2 internalization after NMDA treatment in neurons in which FAIM-L was silenced. Strikingly, GluA2 significantly enhanced internalization even in the absence of NMDA treatment, thereby pointing to the participation of FAIM-L in the regulation of AMPAR trafficking in basal conditions (see +shRNA-FAIM-L; Fig. 3C,E). Additionally, as expected, the induction of GluA2 internalization by NMDA was higher than in the control condition (Fig. 3A–E), resembling a shRNA-XIAP-like phenotype (Fig. 2E,F,I). These observations strongly support the notion that FAIM-L contributes to the regulation of synaptic plasticity.

### FAIM-L overexpression affects synaptic transmission

**Figure 4:**
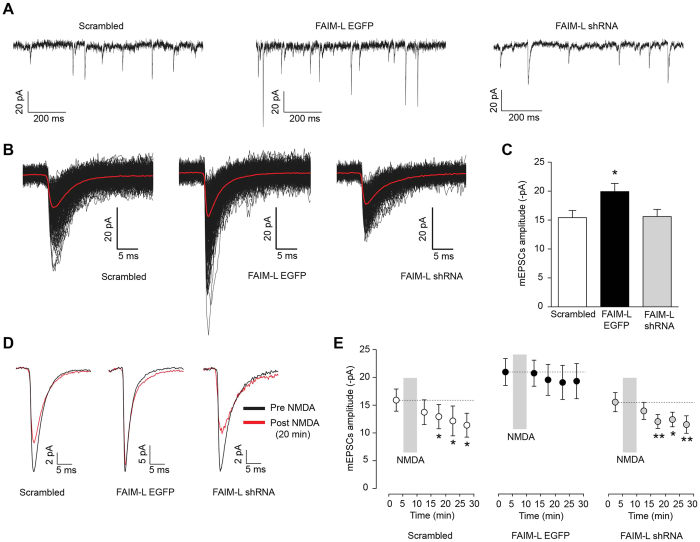
FAIM-L overexpression blocks LTD induction. (**A**) Representative whole-cell recordings of AMPAR-mediated miniature excitatory postsynaptic currents (mEPSCs) of 1-second duration from cultured hippocampal neurons (15–17 DIV) infected with scrambled (left trace), FAIM-L EGFP (middle) and FAIM-L shRNA (right). Membrane potential was held at −60 mV. (**B**) Example of averaged mEPSCs (red lines) from 5-min of the recordings shown in (**A**) This example neuron infected with scrambled (left traces) exhibited a mean mEPSC amplitude of −15.66 pA (average of 341 miniature events) while the neuron overexpressing FAIM-L (right traces) had a mean mEPSC amplitude of −20.48 pA (average of 500 miniature events). Left panel shows mean mEPSC amplitude for the neuron shown in A with FAIM-L shRNA, which display an amplitude of 15.05 pA (average of 198 events). Individual mEPSCs for both recordings are shown in black. Note that larger events are found in the neuron infected with FAIM-L-EGFP. (**C**) AMPAR mEPSC amplitude was increased in FAIM-L-infected cells (−19.93 ± 1.41 pA n = 18) compared with scrambled shRNA (−15.43 ± 1.24 pA n = 16; p = 0.0237, Student t-test). (**D**) Example of averaged mEPSCs response before NMDA application (black traces) and 20 min after NMDA application (red) for the same hippocampal neuron. After recording mEPSCs in baseline conditions (pre-NMDA), 50 μM NMDA was bath applied during 5 min and mEPSCs were recorded consecutively. LTD process was evident in scrambled shRNA and FAIM-L shRNA groups after 20 min of NMDA bath application, while the amplitude of mEPSCs in FAIM-L overexpressing cells was not significantly decreased 20 min after NMDA application. Traces shown are the average of mEPSCs from a 5 min period. Miniature events are scaled to pre-NMDA for comparison purposes. (**E**) Time course of mEPSCs amplitude after NMDA (50 μM) application for scrambled (white circles; n = 8), FAIM-L (black circles; n = 6) and FAIM-L shRNA (grey circles; n = 10). After a 5 min baseline period of mEPSCs recording, NMDA was applied to bath in the absence of TTX or any other blocker for 5 min. Subsequently, miniature events were recorded again in extracellular solution containing blockers during 20 min. LTD was apparent 10, 15 and 20 min after NMDA in scrambled and FAIM-L shRNA groups (*p < 0.05, **p < 0.01; paired Student *t*-test *vs.* baseline period) while no significant LTD was observed even after 20 min in neurons overexpressing FAIM-L (middle panel).

To study the role of FAIM-L in synaptic transmission, FAIM-L was overexpressed in neuronal cultures and measured the AMPAR–mediated miniature excitatory postsynaptic currents (mEPSCs) in the whole-cell configuration. This functional approach allowed us to determine whether synaptic responses are altered in pyramidal neurons overexpressing FAIM-L compared with neurons expressing endogenous FAIM-L levels (scrambled group). In order to detect mEPSCs, recordings were carried out in the presence of TTX in the extracellular solution to block spontaneous evoked transmission. D-AP-5 and picrotoxin were also present in the solution to block NMDA and GABA_A_ receptors. Membrane potential was held at −60 mV during mEPSCs acquisition. We found that AMPAR-mediated mEPSCs amplitude was increased in hippocampal neurons overexpressing FAIM-L (Fig. 4A–C) compared with both scrambled and FAIM-L shRNA groups. Control neurons (scrambled) displayed average amplitude of −15.43 ± 1.24 pA (n = 16), while FAIM-L overexpressing neurons showed increased mEPSCs amplitudes (−19.93 ± 1.41 pA; p = 0.0237 compared to scrambled group; n = 18; Fig. 4C). Silencing of FAIM-L (FAIM-L shRNA) did not alter mEPSCs compared with scrambled group (−15.62 ± 1.22 pA; p = 0.9120; n = 15).

Our data suggest this DR antagonist modulates synaptic transmission. Li and co-workers^6^ discarded the participation of caspase activation in synaptic transmission, and therefore the mechanism through which FAIM-L exerts its function in this context remains elusive. Nevertheless, these observations coincide with the effect on AMPAR internalization observed in the FAIM-L-transduced hippocampal neurons, where the over-expression of this protein resulted in greater internalized AMPAR in non-stimulated neurons (Fig. 2C–I).

### Overexpression of FAIM-L blocks LTD

To study the role of FAIM-L in long-term depression LTD in pyramidal neurons (Fig. 4D,E) we measured mEPSCs amplitude before and after a treatment with NMDA (50 μM during 5 min), a well-established protocol to induce chemical LTD (chem-LTD) either in the CA1 region of hippocampus and in hippocampal neurons in culture^15^,^16^. Miniature events were recorded in 5 min periods for a total of 20 min after NMDA stimulation. NMDA application produced a significant LTD as seen as a decrease in mEPSC amplitude as early as 10 min post-NMDA in scrambled neurons (−15.92 ± 2.00 pA baseline pre-NMDA vs. −12.94 ± 2.18 pA post-NMDA; p = 0.0326; paired student t-test; n = 8; Fig. 4D,E) and also in FAIM-L shRNA (−15.65 ± 1.72 pA baseline pre-NMDA vs. −12.16 ± 1.26 pA post-NMDA; p = 0.0022; paired student t-test; n = 10; Fig. 4.D,E). LTD was even more pronounced at 20 min for scrambled (−11.40 ± 2.15 pA; n = 8; p = 0.0115 paired Student *t*-test) and FAIM-L shRNA (−11.62 ± 1.57 pA; n = 10; p = 0.033). However, no LTD was observed in neurons overexpressing FAIM-L GFP at any time point (−21.08 ± 2.42 pA before NMDA vs. −20.87 ± 2.35 pA, −19.66 ± 2.79 pA, −19.20 ± 3.07 pA and −19.43 ± 3.16 pA at 5, 10, 15 and 20 min post NMDA respectively; p > 0.05 for all time points vs. pre-NMDA; paired Student t-test; n = 6; Fig. 4D,E), indicating that increased levels of FAIM-L in neurons preclude long-term depression changes in mEPSCs amplitude associated with NMDA activation. Our data support the participation of FAIM-L in synaptic transmission and in the modulation of LTD.

### FAIM-L regulates axonal degeneration

During development, neurotrophins are required for cell survival, and they also contribute to neurite growth and maintenance. The action of these molecules results in an overproduction of neural connections that are later removed to form the correct patterns of connectivity. Both neuronal loss and selective developmental axonal degeneration contribute to the adjustment of neuronal connections. Although caspases mediate neuronal apoptosis, they also participate in axonal degeneration^3^. XIAP regulates caspase activation and, as expected, also participates in this process^13^. Since we observed that FAIM-L-promoted XIAP protein stabilization participated in the regulation of a non-apoptotic function of XIAP and caspases such as the LTD, we sought to analyze whether FAIM-L-mediated XIAP stabilization is also involved in axonal degeneration.

**Figure 5:**
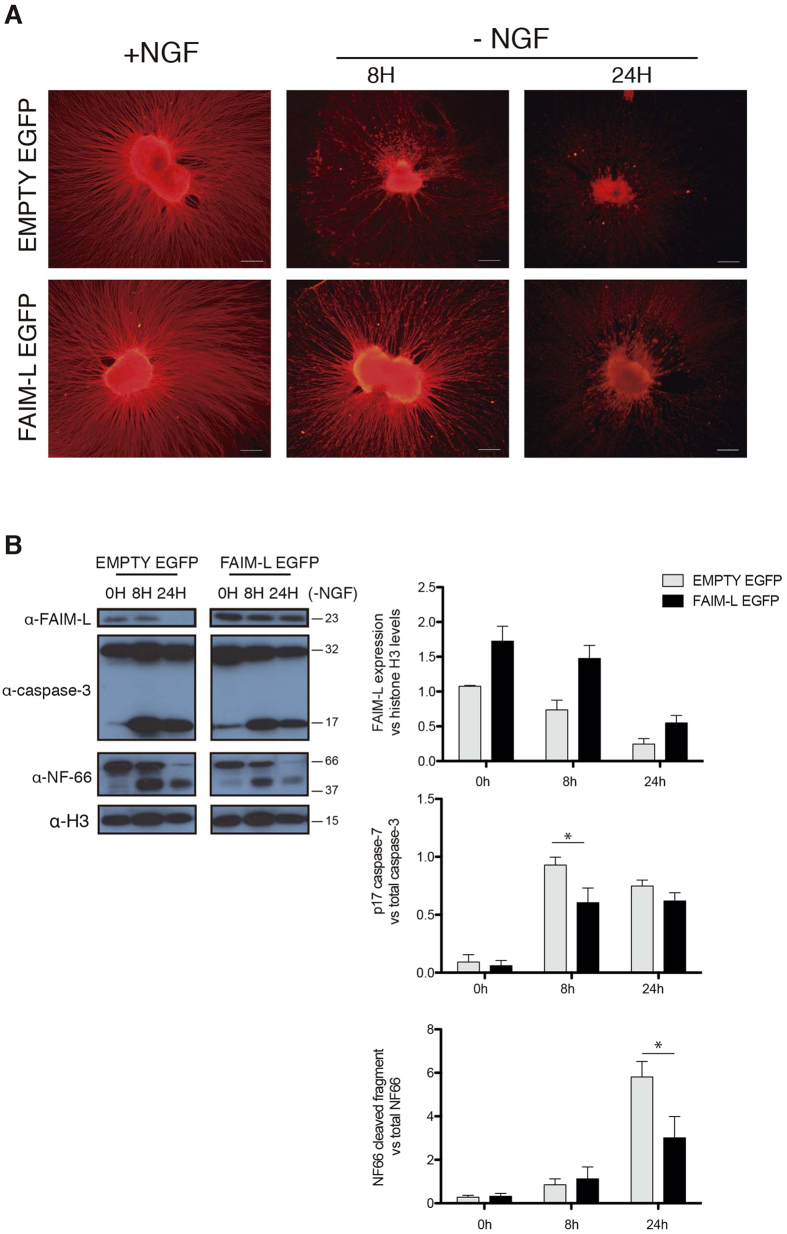
FAIM-L is downregulated in NGF-deprived DRGs and its overexpression impairs axonal DRG degeneration induced by NGF withdrawal. DRG explants were plated in presence of lentiviral vectors carrying EMPTY-EGFP or FAIM-L-EGFP constructs. At DIV 2, DRG explants were subjected to NGF withdrawal. (**A**) at indicated times axonal integrity was assessed by immunocytochemistry against βIII-Tubulin. Representative pictures for EMPT-EGFP- and FAIM-L-EGFP-infected DRGs are shown. Scale bar 200 μm. (**B**) FAIM-L expression, caspase-3 activation, and NF-66 degradation were assessed by Western blot. Histograms show FAIM-L, caspase-3 p17 fragment, and NF-66 cleaved fragment relative levels quantification to Histone H3, total caspase-3 and total NF-66, respectively, from three independent experiments in the indicated conditions. Data are represented as the mean ± standard error of the mean (SEM). Two-way ANOVA test followed by Bonferroni *post-hoc* test was used to calculate significant levels between the indicated groups. *p < 0.05.

First, to address this question we took advantage of a model of axonal degeneration in DRG explant cultures. We dissected out the explants and infected them with the lentiviral vectors for EMPTY-EGFP or FAIM-L-EGFP. Axons were left to grow for 2 DIV and then subjected to NGF deprivation (Fig. 5A). Strikingly, FAIM-L levels in the explants were reduced under these conditions (Fig. 5B). Moreover, FAIM-L overexpression partially protected the explant axons from degeneration after 8–24 h of NGF withdrawal (Fig. 5A).

Many lines of evidence have been published in the field reporting that caspase-3 is involved in axonal degeneration related to axonal pruning during development^3^,^4^,^5^. Indeed, XIAP regulates this process through caspase-3 inhibition. Thus, we addressed whether FAIM-L overexpression interferes with caspase-3 activation during axonal degeneration. Infected explants were deprived of NGF for 8 and 24 h and caspase-3 activation was assessed by Westrn blot. FAIM-L overexpression reduced the generation of the caspase-3 active form p17 at 8 h post-deprivation (Fig. 5B). It has been reported that developmental prompted axonal degeneration involves the activation of calpain by caspase-3 degradation of its inhibitor calpastatin. Therefore, one of the characteristic features of pruning is the appearance of degraded substrates of calpains such as neurofilament 66 (NF-66)^5^. Consistent with the activation of caspase-3 observed in the explants exposed to NGF withdrawal, we detected fragmented forms of NF-66. In this context, the overexpression of FAIM-L induced a reduction in the caspase-3 active fragment at 8 h and a decrease in NF-66 processing at 24 h compared to EMPTY-EGFP- infected explants (Fig. 5B).

### Endogenous XIAP is required for FAIM-L regulation of axonal degeneration

**Figure 6:**
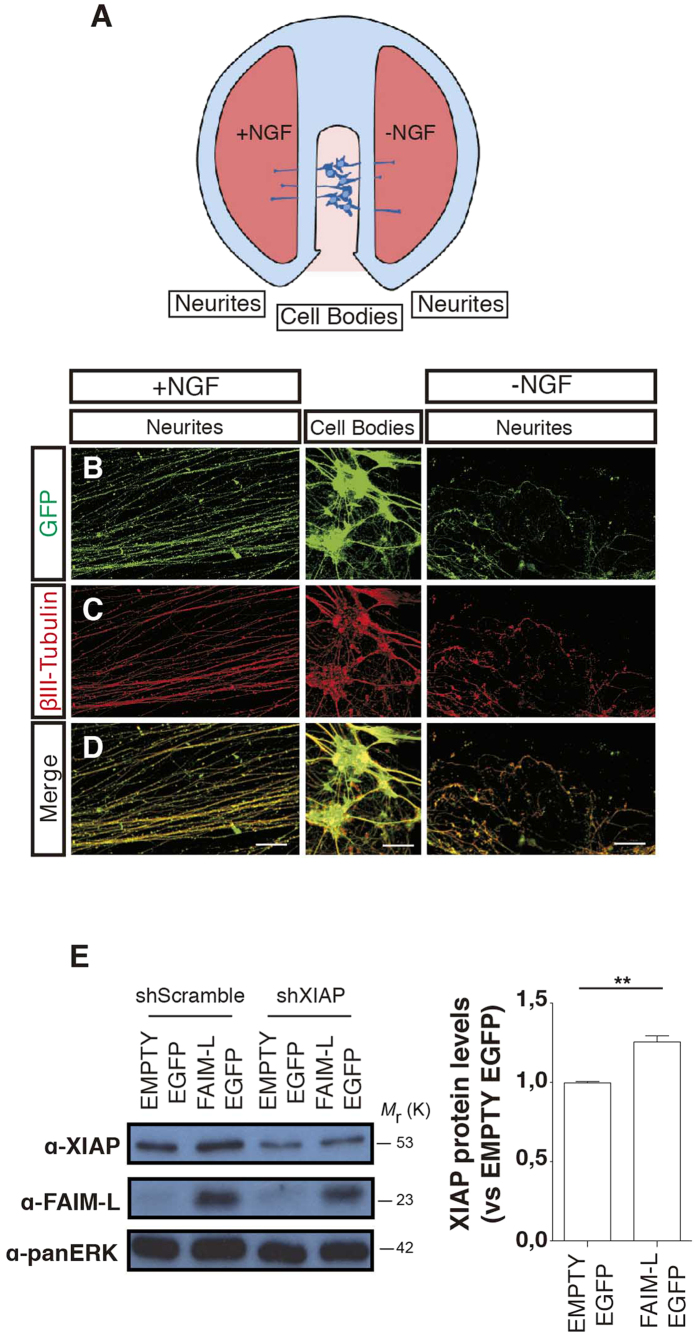
FAIM-L stabilizes XIAP protein levels in DRG neurons. (**A**) Schematic representation of a Campenot chamber. Cell bodies and axonal compartments were subjected (−NGF) or not (+NGF) to NGF withdrawal for 24 h. (**B**–**D**) panels are representative confocal images of the DRG neurons cultured in a Campenot chamber. (**E**) DRGs neurons were infected with either EMPTY-EGFP or FAIM-L-EGFP vectors. Protein levels of XIAP and FAIM-L were determined by Western blot 48 h after infection with overexpressing and silencing vectors. In the scrambled shRNA condition, the intensity of the bands relative to the control (“EMPTY EGFP”) was quantified using ImageJ software. Data are represented as the mean ± SEM of three independent experiments. Student’s *t*-test was used to calculate significant levels between the indicated groups. **p < 0.01. Scale bar 50 μm.

**Figure 7:**
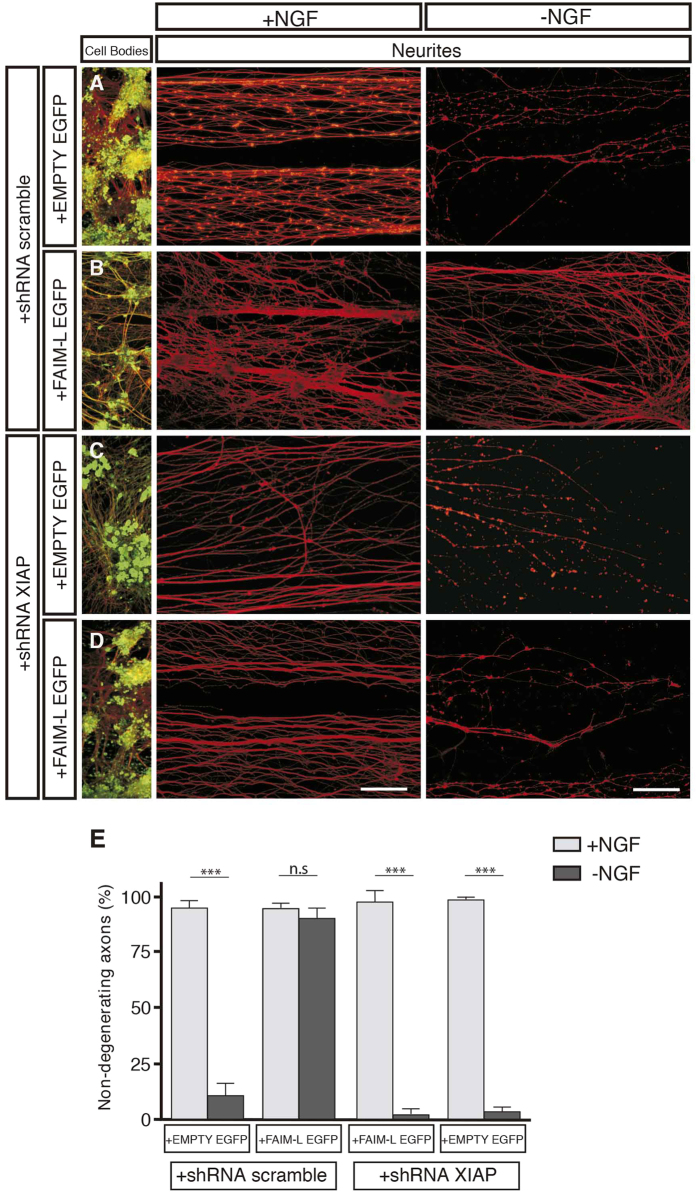
FAIM-L regulates axon degeneration *in vitro* through endogenous XIAP stabilization. (**A**–**D**) Dissociated DRG neurons were plated in Campenot chambers, and distal compartments were filled with medium containing 75 ng/ml human NGF. After double infection with shRNAs (scrambled or XIAP) and overexpressing vectors (EMPTY or FAIM-L), local axonal degeneration was induced in one of the axonal compartments by medium replacement containing sheep anti-NGF 1:50. Cultures were fixed after 24 h and processed for βIII-Tubulin (red) and GFP (green) immunofluorescence. (**E**) Histogram representing the percentage of non-degenerating axons in (**A**–**D**) (mean and SEM, n = 4 replicates). Scale bar 50 μm. Two-way ANOVA test followed by Bonferroni *post-hoc* test was used to calculate significant levels between the indicated groups. ***p < 0.001.

**Figure 8:**
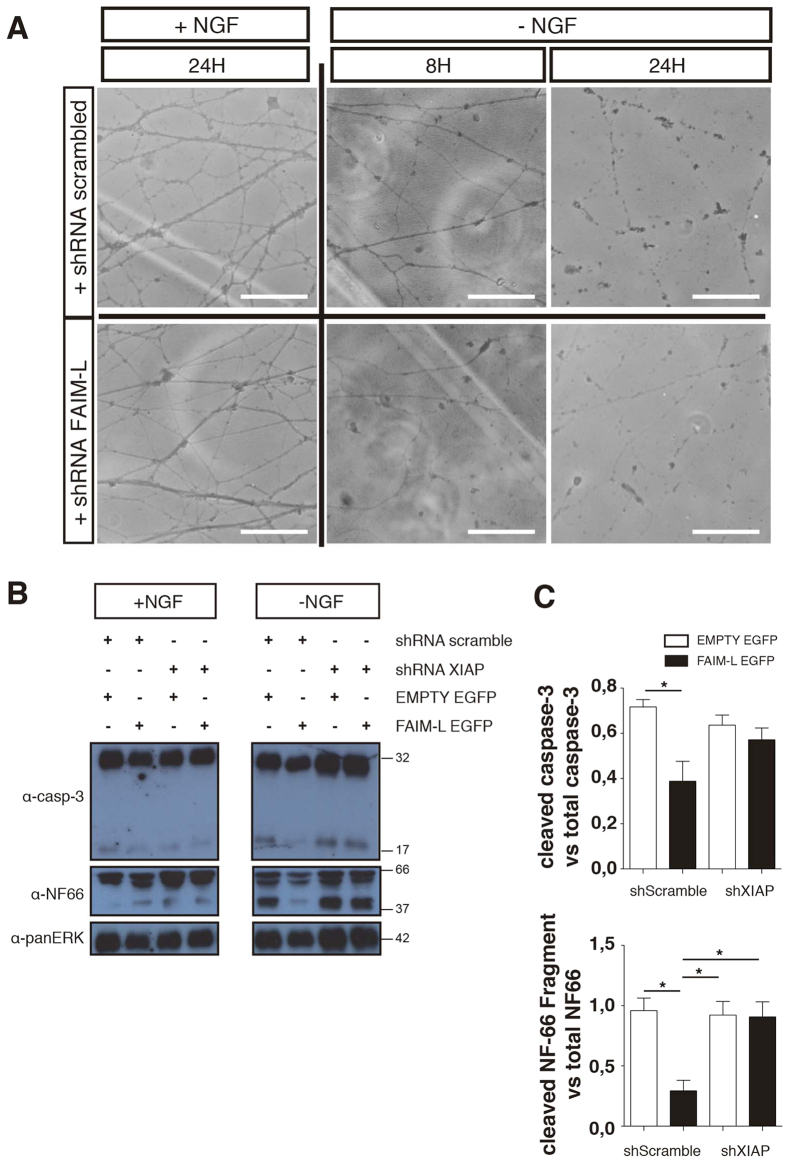
Endogenous FAIM-L participates in axonal degeneration in DRG neurons. (**A**) Dissociated DRG neurons were plated in Campenot chambers, and distal compartments were filled with medium containing 75 ng/ml human NGF. After infection with shRNAs of scrambled or FAIM-L, local axonal degeneration was induced in one of the axonal compartments, as described in materials and methods. Representative confocal images obtained at the indicated times after NGF deprivation are shown. Images are representative of three independent experiments. Scale bar 10 μm. (**B**) DRG cultures were infected with the different lentiviral vectors as indicated, and NGF was withdrawn for 24 h. Caspase-3 (pro-form p32), cleaved caspase-3 (active form p17) and NF-66 levels were assessed at the indicated times by Western blot. ERK was used as loading control. (**C**) Histogram representing active caspase-3 levels relative to total caspase-3 and NF-66 cleaved fragment relative to total NF-66 in each condition 24 h after NGF withdrawal. Data are represented as the mean ± SEM of three independent experiments. One-way ANOVA with Tukey’s multiple comparison post-hoc test was used to calculate significant levels between the indicated groups. *p < 0.05. **p < 0.01.

To further characterize FAIM-L modulation of axonal degeneration, we used Campenot chamber cultures. In these cultures, neurons are seeded in compartmented (Campenot) chambers that allow the establishment of two separate fluid environments. Briefly, DRGs are placed in a central chamber containing NGF, and axons grow inside the lateral chambers (Fig. 6A). Due to limited fluid exchange between the chambers, it is possible to achieve local neurotrophin deprivation that affects only axons, while cell bodies continue to be sustained by NGF (Fig. 6A–D). To determine the role of the FAIM-L/XIAP axis in axonal pruning, DRG neuron bodies were transfected with XIAP-shRNA or scrambled-shRNA and FAIM-L-EGFP or EMPTY-EGFP overexpression constructs (Figs 6E and 7A–E). In agreement with what we previously described in cortical neurons^2^ and the results reported above for hippocampal neurons (Fig. 1B), FAIM-L overexpression promoted the stabilization of XIAP protein levels in DRG neurons (Fig. 6E). When DRG underwent local NGF deprivation, FAIM-L overexpression prevented the axonal degeneration observed after 24 h of NGF withdrawal in control DRG neurons (Fig. 7A,B,E). To test the relevance of FAIM-L-induced XIAP protein stabilization for the capacity of FAIM-L to prevent axon degeneration, we performed the same experiment in the presence of XIAP shRNA (Fig. 7C–E). XIAP down-regulation abolished FAIM-L blockade of axon degeneration, thereby demonstrating that XIAP participates in the effect of FAIM-L on the regulation of axonal degeneration (Fig. 7E). Accordingly, the removal of endogenous FAIM-L levels resulted in faster degeneration when axons were exposed to NGF withdrawal (Fig. 8A). Therefore, our observations in the Campenot chambers support the idea that FAIM-L modulates axonal degeneration in physiological conditions by stabilizing XIAP.

Finally, we determined the status of caspase-3 activation and NF-66 degradation by Western blot. The presence of FAIM-L detected after 24 h of NGF depletion resulted in a reduction of the p17 fragment of caspase-3 and a blockade of NF-66 degradation (Fig. 8B,C). Consistent with our observations regarding axon degeneration, XIAP silencing was sufficient to block FAIM-L inhibition of caspase-3 activation and cleavage of NF-66 (Fig. 8B). These findings further support the notion that FAIM-L exerts its effect by stabilizing endogenous XIAP levels.

All together our data corroborate that the FAIM-L/XIAP/caspase axis is involved in non-apoptotic, neuronal physiological processes, such as axonal pruning and synaptic plasticity.

## Discussion

Here we provide evidence that the DR antagonist FAIM-L regulates axon degeneration and synaptic plasticity by controlling XIAP protein levels. We recently characterized the mechanism underlying the role of the FAIM-L/XIAP axis in neuronal protection against apoptosis. We described that FAIM-L interacts with XIAP through its IAP-binding motif and impairs its auto-ubiquitinylation and proteasomal degradation, subsequently inhibiting caspase activation. The inhibition of XIAP degradation protects neurons against Fas-induced apoptosis^2^. In the present work, we report other functions of the FAIM-L/XIAP axis in a neuronal physiological context that can be added to the previously described role of apoptosis control.

Caspases have been widely related to the resolution of apoptotic cell death^17^. However, these molecules are also activated in neuronal processes that are no longer functional and are marked for elimination^18^,^19^. In this context, it is reasonable to hypothesize that caspases exert functions that do not depend on cell death induction. It is particularly relevant that FAIM-L, an antagonist of apoptosis, is selectively expressed in neurons. As discussed above, caspases are involved in axonal pruning^3^,^4^,^5^ and in the regulation of synaptic plasticity^6^,^7^,^8^.

Mitochondria are well-suited to play a role in synaptic plasticity as this phenomenon modulates their distribution, morphology and motility^15^. Mitochondria in dendrites take up calcium after synaptic stimulation^16^, and this uptake in turn promotes the release of pro-apoptotic factors from these organelles^20^,^21^. Thus, mitochondria are crucial for LTD induction. Li and colleagues reported that the induction of LTD caused by the reduction of AMPA receptor exposure in the membrane is regulated by caspase activity^6^. From the first identification of caspase involvement in this process, most apoptotic members of the intrinsic pathway have been found to correlate with synapse weakening^6^,^7^,^8^. In contrast, proteins that negatively regulate caspase activation, such as XIAP, impede LTD^6^. Our results support all these findings since the silencing of FAIM-L expression in hippocampal neurons dramatically increased the induction of GluA2 internalization in response to NMDA treatment, as observed in XIAP-silenced cells. In agreement with these data, when XIAP protein levels were stabilized by overexpressing FAIM-L, hippocampal neurons did not exhibit significant GluA2 internalization levels after NMDA treatment compared to non-treated neurons and, accordingly, they did not show LTD induction after NMDA treatment.

Nevertheless, our data not only describes the involvement of FAIM-L in the regulation of synaptic plasticity process LTD, but the participation of this DR antagonist in synaptic transmission. We only could detect that FAIM-L overexpression made the neurons prone to a more excited phenotype, as there was an increase in the amplitude of mEPCS, thereby indicating a perturbation of the correct function of the pre- and the post-synaptic synapse in this condition (Fig. 4A,B,C). This is the first time that a DR antagonist has been linked to the modulation of synaptic transmission. The involvement of FAIM-L modulation of XIAP in the mechanism underlying this process is still unknown. There is no clear evidence pointing to XIAP as a modulator of synaptic transmission, and the mediation of caspases in this process has been discarded^6^. Thus, more experiments are required to elucidate whether FAIM-L performs this function through the stabilization of XIAP.

XIAP interacts and inhibits the effector caspases-3 and -7 and the initiator caspase-9^9^,^10^,^11^,^12^. The physiological relevance of XIAP regulation of apoptosis has been related to the modulation of innate immunity, inflammation, and oncogenesis^22^,^23^; however, there is little evidence of its relevance in the CNS. During the development of the nervous system in *Drosophila*, the XIAP homolog DIAP-1 plays a key role in the regulation of caspase activity. DRONC caspase is constitutively active in fly neurons, and therefore DIAP1 is required to restrain its activity during neuronal growth. The inactivation of DIAP-1-mediated DRONC inhibition via DIAP-1 proteasomal degradation is crucial for the reshaping of dendritic arbors on ddAc sensory neurons during morphogenesis^24^. Unsain and collaborators demonstrated that the XIAP-caspase loop that regulates morphogenesis is phylogenetically conserved from flies to mammals^13^. Given our results, we propose that FAIM-L modulates this loop through the stabilization of XIAP. We observed that the overexpression of this DR antagonist not only reduces the axonal degeneration induced by NGF withdrawal but also impairs caspase-3 activation, which is consistent with the inhibition of XIAP degradation. Accordingly, cleavage of NF-66, a target of caspase-3 activation, is also subjected to interference (Figs 5 and 8).

As mentioned above, XIAP regulation of caspase activity in degenerating axons has been elegantly reported by Dr. Barker’s and Dr. Deshmukh’s labs^4^,^13^. Although both studies report higher caspase-3 activation and axon degeneration in XIAP KO mice, only one of them shows a decrease of XIAP levels after NGF withdrawal^13^. Moreover, in the study where the participation of caspase-3 in axon degeneration was stated, they did not observe any changes in XIAP axonal levels after NGF deprivation^3^. In this regard, we have observed low or not evident XIAP down regulation after NGF withdrawal in DRG explant cultures; therefore, we have been unable to study if FAIM-L overexpression modulates XIAP levels in this context. Nevertheless, we observed a reduction in caspase-3 activation and a reduction in NF-66 degradation when we overexpressed FAIM-L. Taking into account these results, the observation that FAIM-L levels are decreased after NGF depletion, and the data shown in Fig. 8A, where we demonstrate that FAIM-L down-regulation by shRNA induces a faster axon degeneration, we believe that FAIM-L participates actively in this process.

The observation that NGF deprivation of DRG explants is accompanied by a downregulation of FAIM-L suggests that the modulation of the stability of this protein or its expression participates in the degenerating cascade. In this context, little is known about the regulation of FAIM-L expression. We have reported that the MEK/ERK pathway is involved in the upregulation of FAIM-L after NGF-induced PC12 differentiation^1^, and, recently, it has been shown that the neural specificity of its expression is governed by NSR100 splicing factor^25^. However, these observations fall short of explaining the regulation of FAIM-L that occurs in the context of axon degeneration. Thus, it would be of interest to further characterize how this protein is regulated during this process in order to shed light on the mechanism that switches FAIM-L-induced XIAP stabilization on and off.

Taken together, our results indicate that, by regulating XIAP protein levels, FAIM-L plays a critical role in regulating non-apoptotic neural events, including axonal degeneration and pruning, and synaptic plasticity events, such as LTD. Moreover, through a mechanism that requires further clarification, we propose that FAIM-L participates in synaptic transmission.

## Methods

### Plasmids

The plasmids pEIGW-EGFP-FAIM-L and pEIGW-EGFP-EMPTY were used for lentiviral overexpression experiments^26^. And pLVTHM-XIAP-shRNA, pLVTHM-FAIM-L-shRNA or pLVTHM-scrambled-shRNA were used for RNA interference^2^. The efficiency in gene expression manipulation by these vectors was tested in Segura *et al.*^1^, and Carriba *et al.*^27^.

### Lentivirus production

Lentiviruses were produced as described by Segura *et al.*^1^.

### Neuronal Culture

All procedures were performed in accordance with the guidelines approved by the Spanish Ministry of Science and Technology and following the European Community Council Directive 86/609 EEC. All experimental protocols were approved by the Vall d’Hebron Institutional Review Board (CEIC) and the Departament d’Agricultura, Ramaderia, Pesca, Alimentació i Medi Natural of the Generalitat de Catalunya (15/14 CEEA).

### Hippocampal neuron cultures and infection

Hippocampal neuron cultures were prepared from mouse embryos at embryonic day (E) 15-16. Brains were dissected in PBS containing 0.6% glucose, and hippocampi were dissected out. After trypsin (Invitrogen) and DNase treatment (Roche Diagnostics), tissue pieces were dissociated, and 50,000 cells were seeded onto 12-mm diameter coverslips coated with 0.5 mg/ml poly-L-lysine (Sigma-Aldrich). Neurons were cultured for 15–18 days in Neurobasal medium supplemented with B27 (Life Technologies), glutamine, 20 U/ml penicillin and 20 μg/ml streptomycin (Sigma-Aldrich, Barcelona, Spain). The medium was further supplemented by adding 1/5 of the volume with conditioned medium from mature (>14 DIV) hippocampal cultures.

Two consecutive lentiviral infections were performed. XIAP shRNA, FAIM-L shRNA or scrambled infection was performed 3 days before the internalization assay; FAIM-L-EGFP or empty-EGFP lentiviral infection was performed 24 h later. Medium containing viruses was changed 5 h after infection. The size of infected cells did not change under the conditions assayed. The percentage of infected cells (GFP-positive) was around 75%, and the efficiency of both FAIM-L overexpression and XIAP down-regulation was assessed by immunocytochemistry.

### Campenot chamber assay of dorsal root ganglia neurons and posterior infection

The Campenot chamber assay was performed as described^28^, with minor modifications. In brief, poly-L-ornithine and laminin-coated Aclar embedded coverslips (Electron microscopy sciences) were scratched with a pin-rake (Tyler research), and a three-compartment Teflon divider was placed on silicone grease. Dissociated sensory neurons from E13 dorsal root ganglia (DRG) were plated in the central compartment using medium supplemented with 10 ng/ml human NGF. The distal compartments were filled with medium containing 75 ng/ml human NGF. The next day, cultures were treated with 5 μM cytosine arabinoside. On days 5 and 7, cultures were infected with the corresponding lentiviruses expressing scrambled, XIAP or FAIM-L shRNAs and EMPTY EGFP or FAIM-L EGFP overexpression vectors, respectively. To trigger local axonal degeneration, NGF-containing medium from axonal compartments was replaced with medium containing sheep anti-NGF 1:50 (Abcam). Cultures were fixed with 4% paraformaldehyde 24 h after NGF removal and processed for βIII-Tubulin (Covance) (1:4000) and GFP (Life technologies) (1:500) immunofluorescence. Alternatively, cells were collected for Western blot analysis.

### Dorsal root ganglia explant culture

Explants culture were obtained from mouse embryos at E13. Embryos were dissected in L-15 and DRG were dissected out. Explants were plated on 48- or 24-well plates, which had been previously coated with poly-L-ornithine and laminin, for protein and immunochemistry analysis, respectively. After dissection, explants were seeded with medium containing 25 ng/mL NGF and infected with lentiviral vectors expressing EMPTY-EGFP or FAIM-L EGFP. After 8 h, the medium was replaced by medium supplemented with 5 μM cytosine arabinoside. At DIV 2, explants were subjected to NGF withdrawal, and protein and immunochemistry analysis were performed at 8 and 24 h.

### Quantification of Axon Viability

To measure the percentage of non-degenerating axons, double immunostained (βIII-Tubulin and GFP) visible axons were counted at the leading edge of the NGF-deprived compartment, and the value was normalized by the number of axons in the compartment containing NGF. Two-way ANOVA test followed by Bonferroni *post-hoc* test was used to calculate significant levels between the indicated groups.

### GluA2 Internalization Assay and Surface Staining

Internalization assays were performed as described^6^, with minor modifications. Briefly, hippocampal neurons at 15–18 days DIV were incubated with antibodies against the N-terminus of GluA2 (2 μg/ml, mouse monoclonal, clone 6C4, Millipore) for 30–60 min at 18–20 °C. After brief washing, neurons were either incubated with control medium or medium containing NMDA (50 μM) for 15 min at 37 °C. Subsequently, they were fixed for 5 min at room temperature in paraformaldehyde (4%)/sucrose (4%) without permeabilization. Surface-remaining antibody-labeled receptors were visualized by means of a 1-h incubation with saturated Cy5-secondary antibody (15 μg/ml, Jackson ImmunoResearch). Neurons were permeabilized for 2 min with methanol (−20 °C), and internalized antibody-labeled GluA2 was detected by a 1-h incubation with Alexa 568-conjugated secondary antibody (1 μg/ml, Invitrogen). Simultaneously, infected GFP-fluorescent neurons were stained with antibodies against GFP (5 μg/ml, Invitrogen, rabbit polyclonal). Thus, the GFP fluorescence of infected neurons was enhanced by a 30 min incubation with Alexa 488-conjugated secondary antibodies (4 μg/ml, Invitrogen). After staining, the coverslips were mounted in Mowiol (Sigma-Aldrich, Barcelona, Spain).

### Image Acquisition and Analysis

Images were acquired using a Leica SP2 spectral confocal microscope with a 63X (NA 1.4) objective. A *z*-stack of images was obtained through various filter channels (Alexa-488, Alexa-568 and Cy5). Typically, 20–25 serial 2D images were recorded at 250-nm intervals. Image acquisition settings were identical in each experiment. FIJI software was used to make 2D projections from the z-stack of images. We measured the total integrated intensity of internalized GluA2 and surface-remaining GluA2 in the same region (which included the cell body and proximal dendrites within 20 μm of the cell body) of infected (GFP-positive) neurons. A threshold was set on the basis of cell-free regions and was kept constant for all conditions in each experiment. Statistical analysis was performed using the One-way ANOVA test followed by Tukey’s or the Newman-Keuls multiple comparison *post-hoc* test.

### Immunocytochemistry and Western Blotting

FAIM-L overexpression, XIAP down-regulation, and their cross-regulation were tested by immunocytochemistry in infected hippocampal primary cultures. Immunocytochemistry was performed using anti-XIAP (1:500; BD Biosciences), anti-FAIM-L (1:500; in house), and rabbit anti-GFP (1:500; Invitrogen), followed by Alexa 568 and Alexa 488-coupled antibodies (1:500; Invitrogen). In DRG neurons, XIAP, FAIM-L, pan-ERK, NF66 and caspase-3 levels were assessed by Western blot using anti-XIAP (BD Biosciences; 1:20,000), anti-FAIM-L (in house; 1:2,000), anti-pan-ERK (BD Biosciences; 1:20,000), anti-NF66 (Covance; 1:10,000), anti-caspase-3 (Cell Signaling; 1:1,000) and Histone H3 (Thermo Scientific; 1:40,000) antibodies.

### Immunocytochemistry and Western Blotting data Analysis and Statistics

Results were analysed using Graphpad Prism, v5.0 (GraphPad Software Inc.). All data were given as mean ± standard error of mean (SEM) and represent the analysis of at least three independent experiments as directed in each specific case. Sample sizes were initially chosen taking into account means and standard deviations from preliminary results obtained from initial exploratory studies. Assuming a Type I error = 5%, and a statistical power ≥0.8 (80%) the sample size for all western blotting was 3, and for the immunocytochemistry experiments was ≥15 cells from 3 independent experiments. Comparisons between two experimental groups were performed using unpaired *t*-test (2-tailed). Experimental groups of more than two were compared using one-way analysis of variance (ANOVA) followed by Tukey’s multiple comparison *post-hoc* test or two-way ANOVA followed by Bonferroni’s *post-hoc* test. Significance was defined as *p < 0.05, **p < 0.01, ***p < 0.001, ****p < 0,0001.

### Electrophysiological Recordings

For the electrophysiological recordings of cultured hippocampal pyramidal neurons (15–17 d.i.v.), coverslips were mounted in a recording chamber placed on the stage of an inverted microscope (Olympus, IX70; Olympus, London, UK or Zeiss, Axiovert 35 M; Oberkochen; Germany). Whole-cell patch-clamp currents were recorded with an Axopatch 200B amplifier – Digidata1322A Series interface board using pClamp10 software (Molecular Devices Corporation, SunnyVale, CA). All recording were performed at room temperature (22–23 °C). Cells were continuously superfused with extracellular solution containing (in mM): 140 NaCl, 3.5 KCl, 10 HEPES, 1 tetraethylammonium chloride (TEA), 20 glucose, 1.8 CaCl_2_, and 0.8 MgCl_2_ (pH 7.4 adjusted with NaOH). To isolate AMPAR-mediated mEPSCs, the following blockers were added to the extracellular solution: 1 μM Tetrodotoxin (TTX; Abcam Biomedical, UK) to block evoked synaptic transmission, 50 μM D-(-)-2-Amino-5-phosphonopentanoic acid (D-AP5; Sigma-Aldrich; St. Louis, MO) to block NMDA receptors, and 100 μM Picrotoxin (Sigma-Aldrich; St. Louis, MO) to block GABA_A_ receptors. Recording electrodes were fabricated from borosilicate glass (PG150T-7.5; 1.5 mm o.d.; 1.16 mm i.d.; Harvard Apparatus Limit, Edenbridge, UK) pulled with a puller P-97 (Sutter Instrument Co., Novato, CA) and had a final resistance of 2.5–4.5 MΩ. Recordings were made using electrodes filled with an internal solution containing (in mM): 116 K-Gluconate, 6 KCl, 2 NaCl, 20 HEPES, 0.5 ethylene glycol tetraacetic acid (EGTA), 2 MgATP, 0.3 Na_2_GTP (pH 7.3 with KOH). Hippocampal pyramidal neurons were chosen in basis of their pyramidal characteristic morphology. Series resistance (Rs) was typically 10–20 MΩ, and was monitored at the beginning and at the end of the experiment. Cells that showed a change in Rs greater than 20% were rejected.

### Chem-LTD Induction

To induce chemical long-term depression (Chem-LTD) in pyramidal neuronal cultures, NMDA was applied at 50 μM for 5 min onto coverslips in physiological recording solution at room temperature. Upon removal of NMDA-containing solution, coverslips were superfused with physiological solution containing blockers, and recordings were performed at time points ranging from 1 to 20 min. Data were grouped in 5-min clusters (1–5 min, 6–10 min, 11–15 and 16–20 min after NMDA application).

### Electrophysiology Data Analysis and Statistics

mEPSCs were filtered at 2 KHz and digitized at 5 KHz. Data were analyzed using IGOR Pro (Wavemetrics Inc., OR, USA), together with Neuromatic (Jason Rothman, UCL). Events were detected using an amplitude threshold of 4–6 pA set according to the baseline current variance. For mEPSC amplitude analysis, only events with a monotonic fast rise (<1.2 ms) and uncontaminated decay were included. When analyzing frequency, any event irrespective of rise time or overlapping decays was included. Statistical analysis was performed using GraphPad Prism version 5.0d for Mac OS X (GraphPad Software, San Diego California USA, www.graphpad.com). Data are presented in the text as the mean ± SEM from n recorded cells and in the figures as bar plots of the group mean, with error bars denoting the SEM. Statistical significance between two groups was examined using the non-parametric Mann-Whitney U test.

## Additional Information

**How to cite this article**: Martínez-Mármol, R. *et al.* FAIM-L regulation of XIAP degradation modulates Synaptic Long-Term Depression and Axon Degeneration. *Sci. Rep.* **6**, 35775; doi: 10.1038/srep35775 (2016).

## References

[b1] Segura M. F. *et al.* The long form of Fas apoptotic inhibitory molecule is expressed specifically in neurons and protects them against death receptor-triggered apoptosis. The Journal of neuroscience: the official journal of the Society for Neuroscience 27, 11228–11241, doi: 10.1523/JNEUROSCI.3462-07.2007 (2007). 17942717 PMC6673028

[b2] Moubarak R. S. *et al.* FAIM-L is an IAP-binding protein that inhibits XIAP ubiquitinylation and protects from Fas-induced apoptosis. The Journal of neuroscience: the official journal of the Society for Neuroscience 33, 19262–19275, doi: 10.1523/JNEUROSCI.2479-13.2013 (2013). 24305822 PMC6618789

[b3] Simon D. J. *et al.* A caspase cascade regulating developmental axon degeneration. The Journal of neuroscience: the official journal of the Society for Neuroscience 32, 17540–17553, doi: 10.1523/JNEUROSCI.3012-12.2012 (2012). 23223278 PMC3532512

[b4] Cusack C. L., Swahari V., Hampton Henley W., Michael Ramsey J. & Deshmukh M. Distinct pathways mediate axon degeneration during apoptosis and axon-specific pruning. Nature communications 4, 1876, doi: 10.1038/ncomms2910 (2013). PMC4183061 23695670

[b5] Yang J. *et al.* Regulation of axon degeneration after injury and in development by the endogenous calpain inhibitor calpastatin. Neuron 80, 1175–1189, doi: 10.1016/j.neuron.2013.08.034 (2013). 24210906

[b6] Li Z. *et al.* Caspase-3 activation via mitochondria is required for long-term depression and AMPA receptor internalization. Cell 141, 859–871, doi: 10.1016/j.cell.2010.03.053 (2010). 20510932 PMC2909748

[b7] Jiao S. & Li Z. Nonapoptotic function of BAD and BAX in long-term depression of synaptic transmission. Neuron 70, 758–772, doi: 10.1016/j.neuron.2011.04.004 (2011). 21609830 PMC3102234

[b8] Han M. H. *et al.* The novel caspase-3 substrate Gap43 is involved in AMPA receptor endocytosis and long-term depression. Molecular & cellular proteomics: MCP 12, 3719–3731, doi: 10.1074/mcp.M113.030676 (2013). 24023391 PMC3861719

[b9] Riedl S. J. *et al.* Structural basis for the inhibition of caspase-3 by XIAP. Cell 104, 791–800 (2001). 11257232 10.1016/s0092-8674(01)00274-4

[b10] Silke J. *et al.* Direct inhibition of caspase 3 is dispensable for the anti-apoptotic activity of XIAP. The EMBO journal 20, 3114–3123, doi: 10.1093/emboj/20.12.3114 (2001). 11406588 PMC150202

[b11] Scott F. L. *et al.* XIAP inhibits caspase-3 and -7 using two binding sites: evolutionarily conserved mechanism of IAPs. The EMBO journal 24, 645–655, doi: 10.1038/sj.emboj.7600544 (2005). 15650747 PMC548652

[b12] Shiozaki E. N. *et al.* Mechanism of XIAP-mediated inhibition of caspase-9. Molecular cell 11, 519–527 (2003). 12620238 10.1016/s1097-2765(03)00054-6

[b13] Unsain N., Higgins J. M., Parker K. N., Johnstone A. D. & Barker P. A. XIAP regulates caspase activity in degenerating axons. Cell reports 4, 751–763, doi: 10.1016/j.celrep.2013.07.015 (2013). 23954782

[b14] Lee S. H., Liu L., Wang Y. T. & Sheng M. Clathrin adaptor AP2 and NSF interact with overlapping sites of GluR2 and play distinct roles in AMPA receptor trafficking and hippocampal LTD. Neuron 36, 661–674 (2002). 12441055 10.1016/s0896-6273(02)01024-3

[b15] Li Z., Okamoto K., Hayashi Y. & Sheng M. The importance of dendritic mitochondria in the morphogenesis and plasticity of spines and synapses. Cell 119, 873–887, doi: 10.1016/j.cell.2004.11.003 (2004). 15607982

[b16] Pivovarova N. B., Pozzo-Miller L. D., Hongpaisan J. & Andrews S. B. Correlated calcium uptake and release by mitochondria and endoplasmic reticulum of CA3 hippocampal dendrites after afferent synaptic stimulation. The Journal of neuroscience: the official journal of the Society for Neuroscience 22, 10653–10661 (2002). 12486158 10.1523/JNEUROSCI.22-24-10653.2002 PMC6758462

[b17] Riedl S. J. & Shi Y. Molecular mechanisms of caspase regulation during apoptosis. Nature reviews. Molecular cell biology 5, 897–907, doi: 10.1038/nrm1496 (2004). 15520809

[b18] Dekkers M. P., Nikoletopoulou V. & Barde Y. A. Cell biology in neuroscience: Death of developing neurons: new insights and implications for connectivity. The Journal of cell biology 203, 385–393, doi: 10.1083/jcb.201306136 (2013). 24217616 PMC3824005

[b19] Hyman B. T. & Yuan J. Apoptotic and non-apoptotic roles of caspases in neuronal physiology and pathophysiology. Nature reviews. Neuroscience 13, 395–406, doi: 10.1038/nrn3228 (2012). 22595785

[b20] Pacher P. & Hajnoczky G. Propagation of the apoptotic signal by mitochondrial waves. The EMBO journal 20, 4107–4121, doi: 10.1093/emboj/20.15.4107 (2001). 11483514 PMC149166

[b21] Szalai G., Krishnamurthy R. & Hajnoczky G. Apoptosis driven by IP(3)-linked mitochondrial calcium signals. The EMBO journal 18, 6349–6361, doi: 10.1093/emboj/18.22.6349 (1999). 10562547 PMC1171698

[b22] Damgaard R. B. *et al.* The ubiquitin ligase XIAP recruits LUBAC for NOD2 signaling in inflammation and innate immunity. Molecular cell 46, 746–758, doi: 10.1016/j.molcel.2012.04.014 (2012). 22607974

[b23] Gyrd-Hansen M. & Meier P. IAPs: from caspase inhibitors to modulators of NF-kappaB, inflammation and cancer. Nature reviews. Cancer 10, 561–574, doi: 10.1038/nrc2889 (2010). 20651737

[b24] Kuo C. T., Zhu S., Younger S., Jan L. Y. & Jan Y. N. Identification of E2/E3 ubiquitinating enzymes and caspase activity regulating Drosophila sensory neuron dendrite pruning. Neuron 51, 283–290, doi: 10.1016/j.neuron.2006.07.014 (2006). 16880123

[b25] Raj B. *et al.* A global regulatory mechanism for activating an exon network required for neurogenesis. Molecular cell 56, 90–103, doi: 10.1016/j.molcel.2014.08.011 (2014). 25219497 PMC4608043

[b26] Moubarak R. S. *et al.* The death receptor antagonist FLIP-L interacts with Trk and is necessary for neurite outgrowth induced by neurotrophins. The Journal of neuroscience: the official journal of the Society for Neuroscience 30, 6094–6105, doi: 10.1523/JNEUROSCI.0537-10.2010 (2010). 20427667 PMC6632611

[b27] Carriba P. *et al.* Amyloid-beta reduces the expression of neuronal FAIM-L, thereby shifting the inflammatory response mediated by TNFalpha from neuronal protection to death. Cell death & disease 6, e1639, doi: 10.1038/cddis.2015.6 (2015). 25675299 PMC4669818

[b28] Campenot R. B., Lund K. & Mok S. A. Production of compartmented cultures of rat sympathetic neurons. Nature protocols 4, 1869–1887, doi: 10.1038/nprot.2009.210 (2009). 20010935

